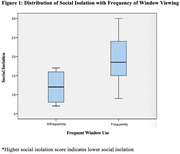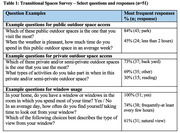# Health Outcomes of Transitional Space Design for Older Adults with Dementia

**DOI:** 10.1002/alz.092405

**Published:** 2025-01-09

**Authors:** Myna Chadalavada, Renee DeCaro, Meltem Karaca, Brenna Hagan, Kristina Morreale, Emily Waskow, Katherine W. Turk, Andrew E. Budson, Diana Anderson

**Affiliations:** ^1^ VA Boston Healthcare System, Jamaica Plain, MA USA; ^2^ Boston University School of Medicine, Boston, MA USA; ^3^ Boston University Alzheimer’s Disease Research Center, Boston, MA USA

## Abstract

**Background:**

The built environment is increasingly recognized as a valid medical intervention, known to affect mental and social health, which can carry consequences for cognitive function. “Transitional spaces” are indoor/outdoor areas designed to foster connections to the surrounding world and boost well‐being including windows, porches, and public parks. Little is known about the space design characteristics that might help improve social and mental health. The goal of this study is to better understand how transitional space engagement impacts health outcomes in those with and without Alzheimer’s disease and related dementias (ADRD).

**Method:**

Surveys were remotely administered to community‐dwelling healthy older adults and those with early‐stage ADRD. A novel built environment survey assessing community and home design features as well as questionnaires measuring loneliness, social isolation, mood, anxiety, and cognitive performance were included. We hypothesized that greater use of residential transitional design features (such as windows) and greater access to outdoor spaces (such as parks) will be associated with positive health outcomes (e.g., lower depression, loneliness).

**Result:**

51 older adults with and without ADRD completed the assessment, consisting of 27 healthy adults and 24 cognitively impaired individuals, ages 55 to 90 (<I>M</I> = 73.9, <I>SD</I> = 6.8). Of the sample, 65% of these individuals reported single family homes, and 78% reported living with someone else. The majority of individuals reported access to community parks (Table 1). Indoor space and window use was also characterized, and individuals who frequently look out the window in their home experienced significantly less social isolation, *t*(41) = 2.92, *p* = .006 (Figure 1). No such relationships were apparent for depression, anxiety, and loneliness. Additional transitional space features and their use will be discussed in further analyses (Table 2).

**Conclusion:**

For older adults who increasingly stay in the home, transitional spaces may allow ways of engaging with the social landscape, promoting benefit, and mitigating effects of isolation. Recognizing the built environment’s role in social connections, mental health and cognition is imperative in designing opportunities for improving the lives of older adults, with and without ADRD.